# Fast scanning chip calorimetry coupled with synchrotron SAXS: an easy-to-use plug-in setup for standard SAXS beamlines

**DOI:** 10.1107/S1600577526010118

**Published:** 2026-10-02

**Authors:** Martin Rosenthal, Akmal Z. Umarov, Goran Ungar, Dimitri A. Ivanov

**Affiliations:** a Department of Chemistry KU Leuven Celestijnenlaan 200F, Box 2404 B-3001Leuven Belgium; b Department of Chemistry Lomonosov Moscow State University Leninskie Gory 1-3 Moscow119991 Russia; c State Key Laboratory for Mechanical Behavior of Materials, Shaanxi International Research Center for Soft Matter, School of Material Science and Engineering Xi’an Jiaotong University Xi’an710049 People’s Republic of China; d School of Chemical, Materials and Biological Engineering University of Sheffield SheffieldS1 3JD United Kingdom; e Institut de Sciences des Matériaux de Mulhouse-IS2M, CNRS UMR 7361, 15 rue Jean Starcky, F-68057Mulhouse, France; ESRF – The European Synchrotron, France

**Keywords:** nanocalorimetry, small-angle X-ray scattering, ultra-long monodisperse *n*-alkane, *in situ* crystallization

## Abstract

A custom-built experimental setup combining *in situ* fast scanning chip calorimetry (FSC) with small-angle X-ray scattering (SAXS) is presented. The setup enables synchronized calorimetric and structural measurements with millisecond acquisition times and heating or cooling rates of up to several thousand kelvin per second. Its capabilities were demonstrated using the ultra-long monodisperse *n*-alkane *n*-C_390_H_782_, a model compound for linear polyethyl­ene. Varying the cooling rate from the melt over nearly five orders of magnitude produces pronounced changes in lamellar thickness, corresponding to different non-integer-folded chain configurations, from approximately the F_5_ form after rapid quenching to F_2_–F_3_ configurations after slow cooling. During subsequent *in situ* heating at 1000 K s^−1^, SAXS revealed a pronounced increase in the average crystalline lamellar thickness from values characteristic of the F_5_ form toward those expected for the F_4_ form, consistent with rapid structural reorganization during the onset of melting. These results demonstrate that significant structural reorganization can occur even under ultrafast heating conditions and highlight the potential of the combined FSC–SAXS approach for probing transient and kinetically controlled structural transformations in polymers and other soft-matter systems.

## Abstract 2

The custom *in situ* fast scanning chip calorimetry–small-angle X-ray scattering (SAXS) setup developed in this work enables synchronized calorimetric measurements and continuous SAXS acquisition with millisecond time resolution during ultrafast thermal protocols. By complementing the calorimetric signal with direct structural information, the method reveals cooling-rate-dependent non-integer-folded chain configurations and follows the evolution of lamellar thickness in the ultra-long monodisperse *n*-alkane *n*-C_390_H_782_ during heating at 1000 K s^−1^.

## 1. Introduction

Over the past three decades, *in situ* synchrotron small- and wide-angle X-ray scattering (SAXS and WAXS) have become established tools for investigating structural transformations in materials, including polymers (Ungar, 1994 ▸; Montano & Oyanagi, 1999 ▸; Masunaga *et al.*, 2007 ▸; Narayanan & Konovalov, 2020 ▸). When combined with controlled heating and cooling stages, these techniques provide direct insight into thermally induced processes such as crystallization, melting, polymorphic transitions, phase separation, and glass-transition-related phenomena (Ivanov *et al.*, 2008 ▸; Christakopoulos *et al.*, 2021 ▸), often under processing-relevant conditions (Li *et al.*, 2025 ▸). Time-resolved measurements enable structural evolution across multiple length scales to be directly correlated with the applied temperature protocol, thereby elucidating the mechanisms governing structure formation and transformation. Furthermore, coupling synchrotron X-ray scattering with differential scanning calorimetry (DSC) allows structural changes to be directly correlated with the corresponding thermal events, making such experiments particularly valuable for understanding and optimizing polymer processing (Ungar & Feijoo, 1990 ▸).

Despite these advantages, conventional heating and cooling stages suffer from intrinsic limitations. Their heating and cooling rates are typically restricted to a few tens of kelvin per minute, which is often insufficient either to suppress thermally activated structural reorganization during heating or to reproduce the rapid thermal histories encountered in industrial polymer processing. Consequently, transient non­equilibrium states and kinetically controlled phase transformations frequently remain inaccessible. This limitation is particularly severe for rapidly crystallizing or reorganizing polymers, in which structural reorganization, crystal growth, and polymorphic transitions may occur on timescales much shorter than those accessible using conventional thermal stages (Heo *et al.*, 2007 ▸).

These limitations can be overcome by fast scanning chip calorimetry (FSC), also referred to as nanocalorimetry, which enables heating and cooling rates of up to 10^3^–10^6^ K s^−1^. In FSC, the sample is deposited directly onto a suspended silicon nitride membrane incorporating integrated resistive heating and temperature-sensing elements. Owing to the extremely low thermal mass of the sensor, precisely controlled ultrafast temperature programs can be applied while simultaneously measuring the heat flow associated with thermal transitions. The technique was pioneered in the mid-1990s by Allen and co-workers, who developed microfabricated calorimetric sensors based on ultrathin non-stoichiometric Si_*x*_N_*y*_ membranes with integrated thin-film electrical circuitry (Lai *et al.*, 1995 ▸). Subsequent developments, particularly by Schick and co-workers, established chip calorimetry as a versatile tool for investigating rapid crystallization, melting, vitrification, physical aging, and homogeneous nucleation in polymers over temperature and time regimes inaccessible to conventional calorimetry (Toda *et al.*, 2016 ▸; Zhang *et al.*, 2024 ▸).

The combination of *in situ* FSC with synchrotron X-ray scattering is therefore highly attractive, as it provides simultaneous structural and calorimetric information under ultrafast thermal conditions (Verma *et al.*, 2023 ▸). Several experimental implementations of this approach have been reported, demonstrating the feasibility of collecting time-resolved WAXS data during rapid heating and cooling (Rosenthal *et al.*, 2014 ▸; Rosenthal *et al.*, 2016 ▸; Lu *et al.*, 2023 ▸; van den Bruinhorst *et al.*, 2023 ▸). These measurements provide access to kinetic pathways that may otherwise be obscured by concurrent crystal growth or structural reorganization and allow thermal histories relevant to industrial polymer processing, including rapid quenching, injection molding, and extrusion, to be reproduced (Melnikov *et al.*, 2016 ▸; Melnikov *et al.*, 2018 ▸; Doblas *et al.*, 2016 ▸). However, the simultaneous acquisition of high-quality SAXS data remains considerably more challenging because of the comparatively weak scattering signal and the stringent requirements for background suppression associated with the calorimetric sensor. Since SAXS provides unique information on nanoscale morphology, including the lamellar long period, lamellar thickness, and hierarchical structural organization, extending FSC experiments to reliable simultaneous SAXS measurements is of considerable importance for polymer science.

The primary objective of the present work was therefore to integrate our custom-designed FSC instrument (Rosenthal *et al.*, 2014 ▸) into a synchrotron SAXS beamline, thereby developing an *in situ* experimental platform capable of performing controlled ultrafast heating and cooling while simultaneously recording calorimetric and SAXS data. Achieving this goal required overcoming several technical challenges, including the miniature dimensions of the calorimetric sensor, accurate synchronization of thermal and scattering measurements, and minimization of parasitic scattering originating from the sensor itself.

To demonstrate the capabilities of the developed setup, we selected as a model system, the monodisperse *n*-alkane *n*-C_390_H_782_, which is the longest monodisperse alkane synthesized to date (Ungar *et al.*, 1985 ▸). Long-chain monodisperse alkanes represent valuable model compounds for polyethyl­ene because they undergo transitions between different integrally folded (IF) and non-integrally folded (NIF) crystal forms, allowing fundamental aspects of polyethyl­ene crystallization and crystal thickening to be investigated in a structurally well defined system. Previous *in situ* studies employing atomic force microscopy (Magonov *et al.*, 2003 ▸) and synchrotron X-ray scattering (Ungar & Zeng, 2001 ▸, Fig. 49) have shown that solution-grown lamellar crystals with chains initially folded in five (F_5_ form) undergo a sequence of thermally induced thickening transitions upon heating, progressively transforming into thicker IF structures and eventually reaching the fully extended form. These well defined structural transformations make *n*-C_390_H_782_ an ideal benchmark material for evaluating the performance of the proposed *in situ* FSC–synchrotron SAXS methodology.

## 2. Experimental setup for *in-situ* fast scanning chip calorimetry

**Figure 1:**
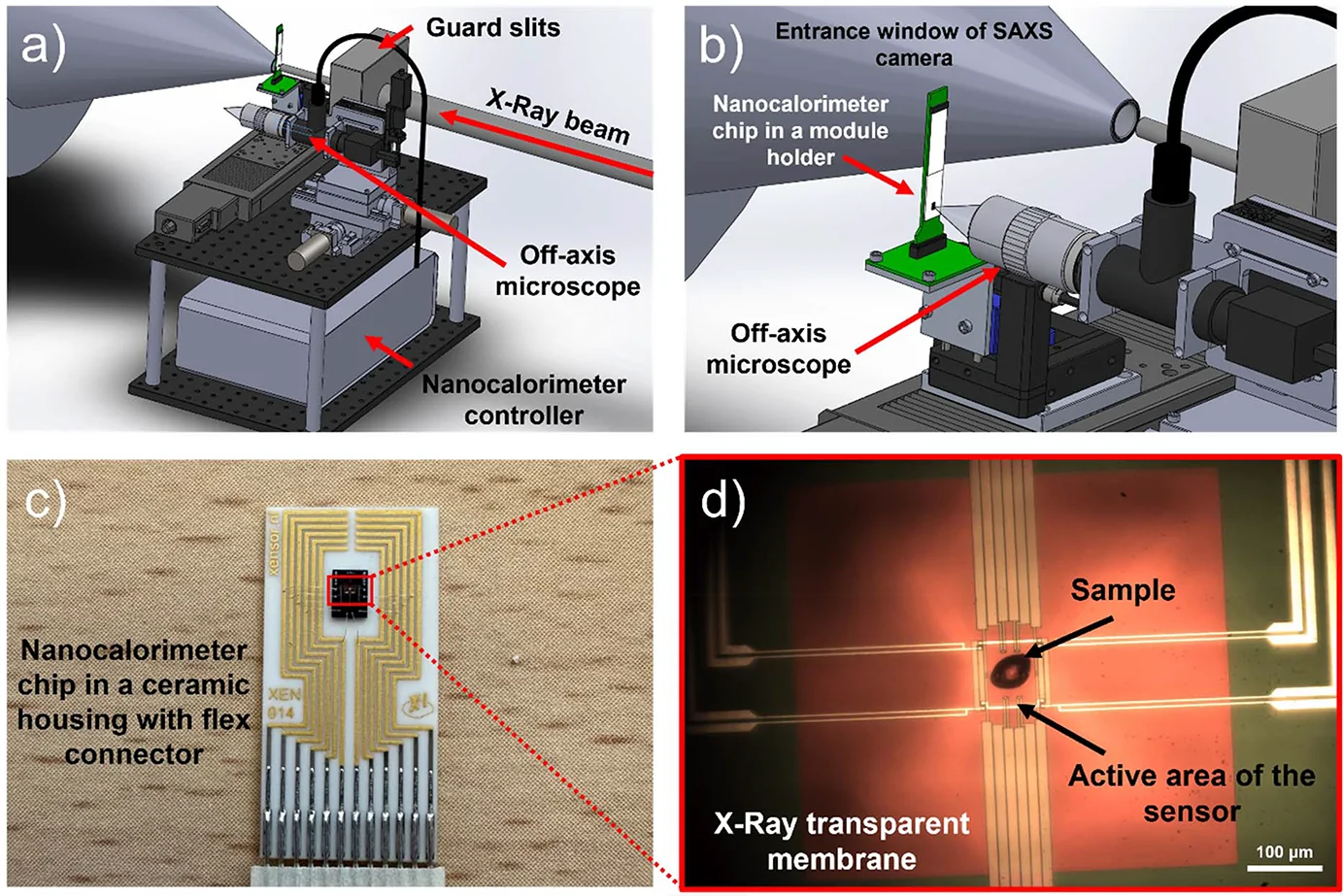
Experimental setup for *in situ* nano­calorimetry–SAXS measurements at the ID02 beamline. (*a*) General view of the setup, showing the nano­calorimeter, guard slits, evacuated SAXS camera, positioning stages, and nanocalorimeter controller. The air gap between the sample and the entrance window of the evacuated SAXS camera was minimized to reduce parasitic scattering from air; mica windows were used for the vacuum components because of their low SAXS background. (*b*) Off-axis optical microscope operated in reflected-light geometry and used for sample positioning. The microscope was first aligned with a test object positioned at a predefined offset from the X-ray beam, thereby establishing an off-axis reference position for subsequent alignment of the nanocalorimetric chip. (*c*) Nanocalorimetric chip mounted on its ceramic support. (*d*) Optical micrograph of the X-ray-transparent active region of the nanocalorimetric chip, as viewed through the microscope objective.

The custom-designed *in situ* fast scanning chip calorimetry setup is shown in Fig. 1 ▸. Since most small-angle X-ray scattering synchrotron beamlines are not equipped with an on-axis optical microscope, accurate positioning of a sample deposited on the active area of a nanocalorimetric chip, typically only about 100 µm × 100 µm, in the X-ray beam is challenging. To overcome this limitation, the setup incorporates an off-axis optical microscope operating in reflected-light geometry [Figs. 1 ▸(*a*) and 1(*b*)]. Preliminary alignment is performed using a reference object consisting of a microparticle of ultra-pure indium positioned in the X-ray beam. The reference object is then translated by a predefined distance away from the beam position, and the off-axis microscope is aligned to it. This procedure establishes a reproducible off-axis optical reference that is subsequently used to position the nanocalorimetric chip relative to the X-ray beam. The microscope, calorimeter, and positioning system are integrated into a single modular unit, facilitating transfer of the setup to other synchrotron SAXS beamlines without major modifications to their experimental endstations.

An additional requirement for SAXS measurements is the minimization of parasitic background scattering. This was achieved by positioning guard slits immediately upstream of the sample and by minimizing the air path around the sample, with approximately 5 mm of air gap upstream and 5 mm downstream between the sample and the entrance window of the evacuated SAXS flight tube [Fig. 1 ▸(*b*)]. Mica was used for the X-ray windows of the vacuum components because of its low background contribution in the SAXS range. A general view of the nanocalorimetric chip mounted on its ceramic support and connected via a flexible electrical connector is shown in Fig. 1 ▸(*c*). A magnified view of the active area of the chip, located on a thin X-ray-transparent Si_*x*_N_*y*_ membrane (van Herwaarden, 2005 ▸), is presented in Fig. 1 ▸(*d*).

The SAXS experiments using the setup described above were carried out at the ID02 beamline of the European Synchrotron Radiation Facility (ESRF) in Grenoble, France. Measurements were performed in transmission geometry using an X-ray photon energy of 12.23 keV. To obtain a beam size of approximately 40 µm × 40 µm at the sample position, the X-ray beam was focused using a toroidal mirror and further collimated by the beamline slit system. The required photon flux of approximately 5 × 10^12^ photons s^−1^ was provided by two phased U21.4 undulators tuned to deliver maximum flux at 12.23 keV (Narayanan *et al.*, 2022 ▸).

The accessible range of the scattering-vector magnitude was 5.0 × 10^−2^ to 2.0 nm^−1^, with *q* = 4πsinθ/λ, where θ is half the scattering angle and λ is the X-ray wavelength. Two-dimensional scattering patterns were recorded using an Eiger2 4M detector positioned at a sample-to-detector distance of 3.0 m. For synchronized *in situ* acquisition of the two-dimensional scattering patterns and the nanocalorimetric signal, a TTL trigger generated by the nanocalorimeter was sent to a multiplexer controlling both the X-ray detector acquisition and the fast X-ray shutter. The *in situ* measurements were performed with an exposure time of 1.9 ms per frame. Further technical details of the synchronization scheme and nano­calorimetric setup can be found in our previous publications (Melnikov *et al.*, 2018 ▸; Rosenthal *et al.*, 2016 ▸).

The background signal originating from the empty nanocalorimetric chip was subtracted from each curve. Data correction, calibration, and azimuthal integration were performed using the *pyFAI* Python library (Ashiotis *et al.*, 2015 ▸).

The SAXS curves were analyzed using the one-dimensional correlation function *K*(*z*) as described by Strobl & Schneider (1980 ▸),
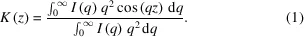
The long period, *L*_p_, and crystalline lamellar thickness, *L*_c_, were extracted from the correlation function using the first subsidiary maximum and the self-correlation triangle, respectively. The fully extended chain length of *n*-C_390_H_782_, *L*_0_, is calculated to be 49.55 nm. For the calculation of the chain configuration, it is noteworthy that in rapidly melt-crystallized *n*-alkanes the chains are oriented perpendicular to the lamellar basal plane, as previously demonstrated by FTIR and SAXS (de Silva *et al.*, 2002 ▸; de Silva *et al.*, 2003 ▸). Therefore, the crystal thickness of an alkane chain comprising *n* segments after folding can be expressed as
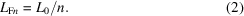


## 3. Results and discussion

To demonstrate the capabilities of the experimental setup, we selected the ultralong monodisperse *n*-alkane *n*-C_390_H_782_ as a model system. This material is particularly well suited for studying polymer crystallization because it exhibits discrete transitions between integer-folded forms (F_2_–F_5_), as described by Magonov *et al.* (2003 ▸). Static measurements performed on samples with masses on the order of 100 ng showed that sufficient SAXS intensity could be obtained with exposure times of approximately 2 ms. This demonstrated the feasibility of *in situ* fast-heating experiments with millisecond time resolution.

In the following sections, we first analyze the lamellar microstructure formed upon cooling from the melt at defined cooling rates, as determined from static SAXS measurements performed at room temperature. We then present the results of an *in situ* heating experiment with continuous acquisition of SAXS patterns.

### 3.1. Influence of cooling rate on the lamellar microstructure of C_390_H_782_

**Figure 2:**
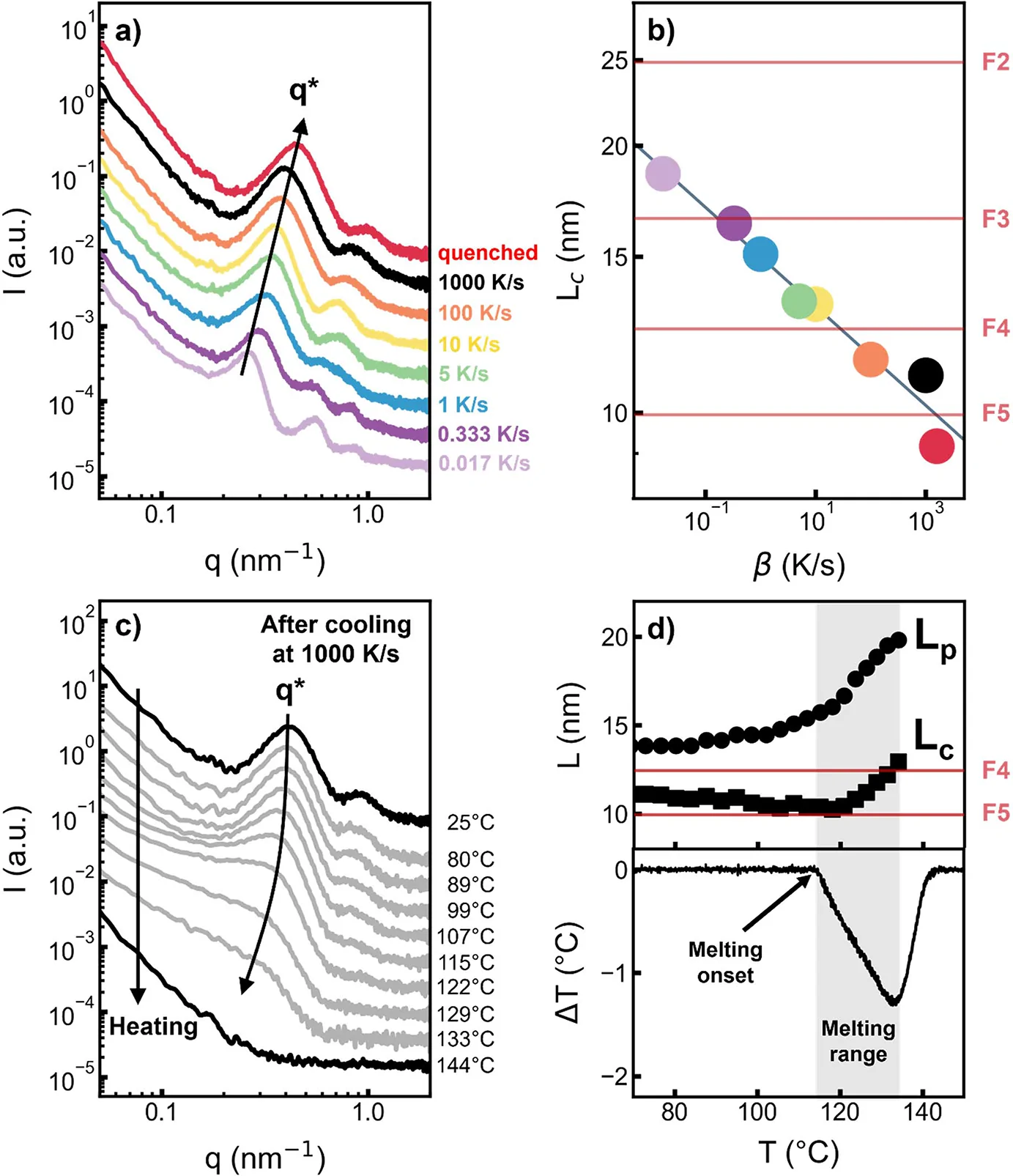
(*a*) SAXS curves of the C_390_H_782_ sample recorded after cooling from the melt at different cooling rates, β. (*b*) Crystalline lamellar thickness, *L*_c_, as a function of β. The red lines indicate the expected lamellar thicknesses of the integer-folded forms F_2_, F_3_, F_4_, and F_5_. (*c*) SAXS curves recorded during *in situ* heating of C_390_H_782_, previously cooled at β = 1000 K s^−1^, using the integrated nanocalorimeter;. (*d*) Temperature dependence of *L*_c_, together with the corresponding nanocalorimetric signal. A baseline correction was applied to the nanocalorimetric signal in order to emphasize the thermal effect associated with the phase transition. The SAXS curves in panels (*a*) and (*c*) are vertically offset for clarity.

The C_390_H_782_ sample positioned on the active area of the nanocalorimeter chip was briefly heated to the melt state at 150°C and subsequently cooled to room temperature at controlled rates ranging from 1 K min^−1^ to more than 1000 K s^−1^. The corresponding room-temperature SAXS curves are shown in Fig. 2 ▸(*a*). The curve denoted as ‘quenched’ corresponds to the sample cooled to room temperature by abruptly switching off the chip heating. At the crystallization temperature of C_390_H_782_, the resulting cooling rate was approximately 3000 K s^−1^.

The position of the main interference peak, q*, strongly depends on the cooling rate, β. With increasing β, the peak systematically shifts toward higher *q*-values [*cf*. Fig. 2 ▸(*a*)], indicating a decrease in the lamellar long period, *L*_p_ ≃ 2π/q*, which represents the average distance between the neighboring lamellae. Most SAXS curves exhibit two orders of the main interference peak, whereas the curves recorded after relatively slow cooling from the melt display three orders, indicating a higher degree of regularity in the lamellar structure. The one-dimensional SAXS correlation functions were used to determine both the crystalline lamellar thickness, *L*_c_, and the long period, *L*_p_ (Fig. S1 in the supporting information). The resulting values are summarized in Table 1 ▸ and plotted as functions of the cooling rate in Fig. 2 ▸(*b*). The value of *F*_avg_ reported in Table 1 ▸ represents the average number of crystalline stems per molecule.

It can be seen that, upon rapid cooling from the melt, the attainable lamellar thickness is consistent with the F_5_ configuration of the alkane. Such thin melt-grown crystals are comparable with the F_5_ crystals obtained from dilute solution (Magonov *et al.*, 2003 ▸). This result demonstrates the ability of the setup to achieve deep quenching through the high cooling rates employed, since crystals grown from the melt are generally thicker than those obtained from solution. As the cooling rate decreases, the resulting lamellar thickness progressively increases, reaching an NIF configuration between F_2_ and F_3_ at the lowest cooling rate of 0.017 K s^−1^. At the same time, the linear crystallinity remains between approximately 0.74 and 0.83 over the entire range of cooling rates, with a tendency toward higher values at lower cooling rates.

### 3.2. Evolution of the lamellar microstructure during fast heating of C_390_H_782_

An *in situ* nanocalorimetric experiment was performed on a sample initially crystallized during cooling at a rate of β = 1000 K s^−1^. In the experiment, the sample was heated to 150°C at 1000 K s^−1^, while SAXS and the nanocalorimetric signal were recorded simultaneously. The evolution of the SAXS curves during heating is shown in Fig. 2 ▸(*c*). At the beginning of the heating ramp, the position of the main interference peak remains nearly unchanged and subsequently shifts toward lower *q*-values. Simultaneously, the peak gradually decreases in intensity and broadens, while the higher-order reflections disappear at the onset of melting, as identified from the nanocalorimetric curve [Fig. 2 ▸(*d*)]. The nanocalorimetric signal exhibits a melting endotherm extending from approximately 115 to 135°C. Melting is completed when the nanocalorimetric signal reaches the maximum of the melting peak, as expected for the present non-power-compensated heat-flux calorimetric configuration.

An important result of the present experiment is that the SAXS data acquired during ultrafast heating retained sufficient quality for quantitative structural analysis. Although the individual exposure time was only 1.9 ms, averaging two consecutive frames was sufficient to obtain an adequate signal-to-noise ratio after subtraction of the empty-chip background. The high-*q* region of the resulting SAXS curves could be reliably extrapolated using Porod’s law, allowing calculation of the one-dimensional correlation functions and extraction of the lamellar structural parameters. Thus, the present measurements demonstrate that millisecond-resolved SAXS acquired during heating at 1000 K s^−1^ can provide not only qualitative information on peak evolution but also quantitatively meaningful micro-structural parameters.

The results of the correlation-function analysis, together with the nanocalorimetric signal, are presented in Fig. 2 ▸(*d*); representative correlation functions are shown in Fig. S1 in the supporting information. Throughout the heating experiment, the long period, *L*_p_, increases monotonically up to melting. In contrast, the crystalline lamellar thickness, *L*_c_, initially remains close to the value characteristic of the F_5_ form and then exhibits a pronounced increase at around 115°C, approaching the value expected for the F_4_ form. The onset of this increase coincides with the onset of melting.

Lamellar thickening during heating has previously been reported for solution-grown C_390_H_782_ crystals by variable-temperature atomic force microscopy (Magonov *et al.*, 2003 ▸) and SAXS (Ungar & Zeng, 2001 ▸, Fig. 49), as well as for linear polyethyl­ene during slow heating (Christakopoulos *et al.*, 2021 ▸). In the present experiment, the increase in the SAXS-derived average *L*_c_ occurs within only about 15 ms, demonstrating that substantial changes in the lamellar structure can take place even at a heating rate of 1000 K s^−1^. The observed evolution is consistent with rapid structural reorganization of the non-integer-folded C_390_H_782_ crystals during the early stage of melting. Possible microscopic mechanisms include lamellar thickening through incorporation of cilia into the crystalline regions and reconfiguration of loose folds near the melting front.

It should be noted, however, that the present SAXS data alone do not allow the thickening of individual lamellae to be distinguished unambiguously from preferential melting of thinner lamellae, which would likewise lead to an increase in the average *L*_c_. Therefore, the observed increase in *L*_c_ is regarded as evidence for rapid reorganization of the lamellar population rather than as an unequivocal demonstration of lamellar thickening. Nevertheless, the results show that significant structural reorganization of C_390_H_782_ can occur on a millisecond time scale under ultrafast heating conditions.

## 4. Conclusions

We have described a custom-built setup that combines *in situ* fast scanning chip calorimetry with small-angle X-ray scattering and enables synchronized static and dynamic measurements with acquisition times on the millisecond scale. This makes it possible to perform *in situ* heating and cooling experiments at rates of up to several thousand kelvin per second while simultaneously monitoring nanoscale structural evolution. It has been shown that the flux provided by the ID02 beamline of approximately 5 × 10^12^ photons s^−1^ in a sub-50 µm × 50 µm beam in combination with an Eiger2 X 4M detector is sufficient to collect SAXS data with an adequate signal-to-noise ratio within such short acquisition times, allowing the calculation of high-resolution autocorrelation functions for detailed analysis of the morphological parameters of the studied materials. In contrast, in previous experiments (Melnikov *et al.*, 2018 ▸; Rosenthal *et al.*, 2016 ▸) where a synchrotron micro-beam setup was used in a WAXS configuration with a microscopic beamstop, the SAXS data are compressed into a few pixels limiting the resolution of the *q*-range binning. The use of much smaller beams of approximately 15 µm × 15 µm in FSC/µWAXS experiments using a comparable photon flux to the ID02 experiment reported here leads to a much higher flux density by more than a factor of four in the µWAXS experiments making the measurements more prone to beam-induced damage in the polymer material, while number of interacting photons with the sample are similar. The capabilities of the setup were demonstrated using *n*-C_390_H_782_, the longest monodisperse *n*-alkane synthesized to date, which serves as a model system for linear polyethyl­ene. By varying the cooling rate from the melt over nearly five orders of magnitude, clear changes between different folded lamellar forms were observed. During rapid *in situ* heating, the temperature evolution of the lamellar structure could be followed directly. In particular, the observed increase in lamellar thickness from values corresponding initially to the F_5_ form toward those expected for the F_4_ form is consistent with structural reorganization occurring at a heating rate as high as 1000 K s^−1^.

These results demonstrate the broad potential of the developed setup for investigating the thermal behavior of polymers and, more generally, soft-matter systems under a wide range of thermal protocols. The combination of millisecond time resolution, ultrafast temperature control, and simultaneous SAXS measurements provides access to transient and kinetically controlled structural states that are difficult to investigate using conventional thermal stages. The ultimate time resolution of such measurements is determined by the balance between photon flux, detector performance, sample scattering intensity, background contribution, and the statistical quality required for quantitative SAXS analysis.

## Supplementary Material

Supporting Figure S1. DOI: 10.1107/S1600577526010118/ok5174sup1.pdf

## Figures and Tables

**Table 1:** Structural parameters of C_390_H_782_ crystallized from the melt at different cooling rates, obtained from the 1D SAXS correlation function analysis

β (K s^−1^)†	*L*_a_ (nm)‡	*L*_c_ (nm)§	*L*_p_ (nm)¶	χ_cr_††	*F*_avg_ ‡‡
Quenched	3.3	9.2	12.4	0.74	5.38
1000	3.0	11.0	14.0	0.79	4.50
100	3.3	11.5	14.8	0.78	4.30
10	3.4	13.3	16.7	0.80	3.72
5	3.5	13.4	16.8	0.79	3.71
1	3.1	15.1	18.2	0.83	3.28
0.333	4.5	16.4	20.9	0.78	3.02
0.017	4.0	18.6	22.6	0.82	2.66

† Cooling rate from the melt.

‡ Amorphous layer thickness, *L*_a_.

§ Crystalline lamellar thickness, *L*_c_.

¶ Long period, *L*_p_. The parameters *L*_a_, *L*_c_, and *L*_p_ were determined from the 1D SAXS correlation function.

†† Linear crystallinity, *L*_c_/*L*_p_.

‡‡ Average number of crystalline stems per molecule, *F*_avg_ = *L*_0_/*L*_c_.

## Data Availability

The raw SAXS data collected at beamline ID02 (ESRF) are available via the ESRF data repository under https://doi.org/10.15151/ESRF-ES-2148348224. Additional data supporting this article are available from the corresponding author upon reasonable request.
