# Supplementary material for: Fast scanning chip calorimetry coupled with synchrotron SAXS: an easy-to-use plug-in setup for standard SAXS beamlines
Source: J Synchrotron Radiat. 2026 Oct 2;33(Pt 6):1801–6. doi: 10.1107/S1600577526010118 (PMC13753866; doi:10.1107/S1600577526010118)
Supplement: Supplementary file 1 [file s-33-01801-sup1.pdf]

JOURNAL OF
SYNCHROTRON
RADIATION

Volume 33 (2026)

Supporting information for article:

Fast Scanning Chip Calorimetry Coupled with Synchrotron Small-Angle X-ray Scattering (SAXS): An Easy-to-Use Plug-In Setup for Standard SAXS Beamlines

Martin Rosenthal, Akmal Z. Umarov, Goran Ungar and Dimitri A. Ivanov

S1. SAXS data treatment and 1D correlation functions

The SAXS intensity was corrected for background and thermal diffuse scattering. At high q, the curves were extrapolated according to Porod's law. At low q, a power-law term was subtracted from I(q).

The 1D SAXS correlation functions were computed and analyzed following the classical approach described in Ref. (Strobl & Schneider, 1980).

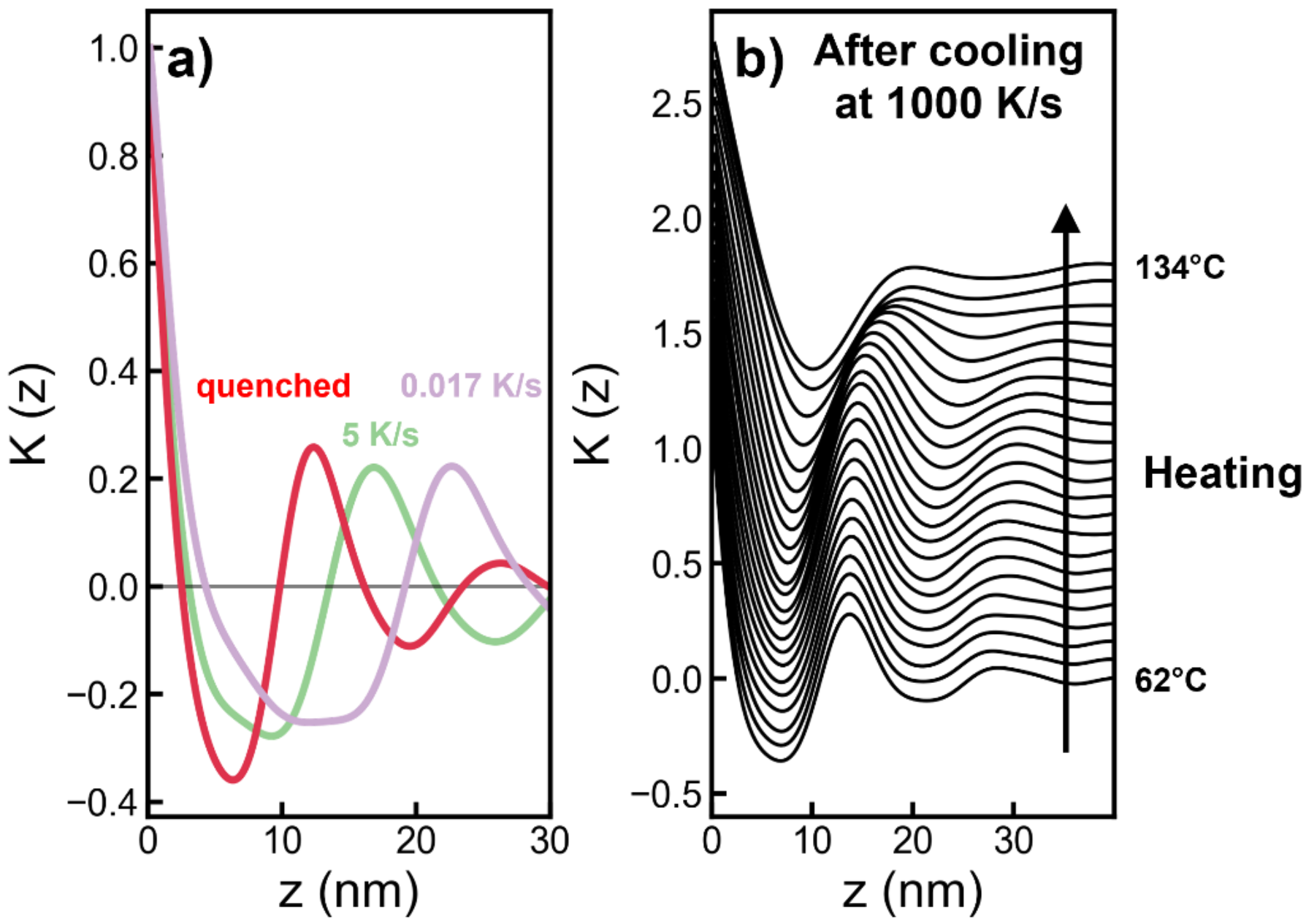

Figure S1 (a) Correlation functions calculated from the SAXS patterns shown in Fig. 2a of the main text and recorded at room temperature for $C_{390}H_{782}$ crystallized from the melt at the indicated cooling rates. (b) Correlation functions calculated from the SAXS patterns shown in Fig. 2c of the main text and recorded during heating of $C_{390}H_{782}$ previously crystallized at 1000 K s^{-1}.
